# Carbamates as Potential Prodrugs and a New Warhead for HDAC Inhibition

**DOI:** 10.3390/molecules23020321

**Published:** 2018-02-02

**Authors:** Kristina King, Alexander-Thomas Hauser, Jelena Melesina, Wolfgang Sippl, Manfred Jung

**Affiliations:** 1 Institute of Pharmaceutical Sciences, Albert-Ludwigs-University Freiburg, Albertstraße 25, 79104 Freiburg im Breisgau, Germany; kristina0keller@googlemail.com (K.K.); alexander.hauser@pharmazie.uni-freiburg.de (A.-T.H.); 2 Institute of Pharmacy, Martin-Luther-University Halle-Wittenberg, Wolfgang-Langenbeck-Straße 4, 06120 Halle (Saale), Germany; jelena.melesina@pharmazie.uni-halle.de (J.M.); wolfgang.sippl@pharmazie.uni-halle.de (W.S.)

**Keywords:** epigenetics, histone deacetylases, HDACs, hydroxamic acids, prodrug concept

## Abstract

We designed and synthesized carbamates of the clinically-approved HDAC (histone deacetylase) inhibitor vorinostat (suberoylanilide hydroxamic acid, SAHA) in order to validate our previously-proposed hypothesis that these carbamates might serve as prodrugs for hydroxamic acid containing HDAC inhibitors. Biochemical assays proved our new compounds to be potent inhibitors of histone deacetylases in vitro, and they also showed antiproliferative effects in leukemic cells. These results, as well as stability analysis led to the suggestion that the intact carbamates are inhibitors of histone deacetylases themselves, representing a new zinc-binding warhead in HDAC inhibitor design. This suggestion was further supported by the synthesis and evaluation of a carbamate derivative of the HDAC6-selective inhibitor bufexamac.

## 1. Introduction

Histone deacetylases (HDACs) are some of the best-studied histone modifying enzymes within the still expanding field of epigenetics. They are validated targets for anticancer therapy with five approved drugs on the market and a growing number of HDAC inhibitors in promising clinical trials [1,2,3,4,5,6,7]. Besides the Sirtuins (class III HDACs) that possess a catalytic mechanism depending on NAD (nicotine adenine dinucleotide), the classical HDACs (classes I, II and IV) are zinc-dependent amidohydrolases. Therefore, all inhibitors of these histone deacetylases feature a zinc-chelating group, which is essential for their inhibitory activity. One of the most commonly-employed zinc-binding groups is the hydroxamic acid moiety, which also is present in the FDA (Food and Drug Administration) approved inhibitors vorinostat (*N*-hydroxy-*N*′-phenyloctanediamide, SAHA), belinostat and panobinostat, as well as in many clinical candidates. Hydroxamic acids are known to be problematic with regard to their pharmacokinetic properties, especially the metabolic stability in vivo is limited. Thus, a reasonable prodrug concept for hydroxamic acids might lead to an improvement of stability, metabolism or cellular uptake of such inhibitors.

In previous work, we were able to show that carbamates might serve as promising prodrugs for hydroxamic acids [8]. Our hypothesis also was picked up and analyzed by Seki and co-workers [9]. They could show that carbamates are potent, but much less toxic inhibitors of the BoNT/A (botulinum neurotoxin A) protease than the parent hydroxamic acid.

For this study, we synthesized carbamates of the FDA-approved HDAC inhibitor SAHA with varying side chains in order to validate our previous findings and to investigate the effect of the different substitution patterns on the stability and inhibition properties. We also synthesized a water-soluble carbamate with a terminal basic moiety, aiming at improved pharmacokinetic properties, which would especially be beneficial for animal studies.

## 2. Results and Discussion

### 2.1. Synthesis

**Scheme 1:**
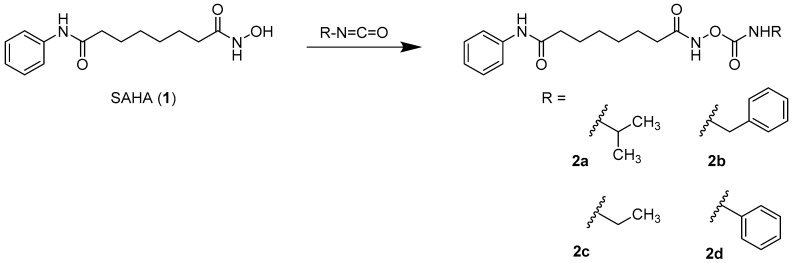
Route of synthesis for the carbamates **2a**–**2d** starting from the parent compound SAHA (1). Reaction conditions: dimethylformamide (DMF), acetone, −15 °C.

**Scheme 2:**
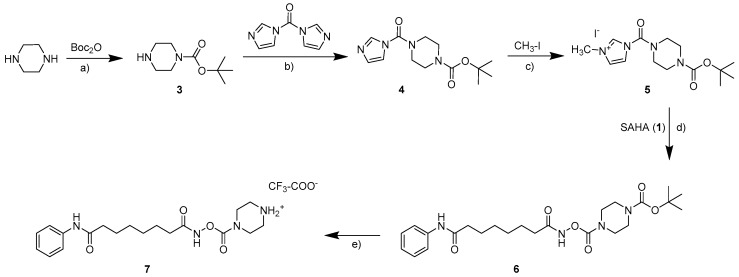
Route of synthesis for the carbamate **7**. *Reaction conditions*: (**a**) dichloromethane, room temperature; (**b**) dichloromethane, 0 °C; (**c**) acetonitrile, 0 °C; (**d**) acetone, dimethylformamide, room temperature; (**e**) dichloromethane, trifluoroacetic acid (TFA), 0 °C.

The parent compound SAHA (**1**, vorinostat; see Scheme 1) was synthesized as described in the literature [10,11]. The synthesis of carbamates starting from hydroxamic acids can in general be realized with isocyanates as monosubstituted transfer reagents or with *N*,*N*-disubstituted carbamoyl chlorides. As carbamoyl chlorides are very unstable and difficult to isolate [12], the synthesis of the carbamates **2a**–**2d** (see Scheme 1) was carried out with differently substituted isocyanates as previously described [8]. As no isocyanates bearing basic moieties were commercially available, for the water soluble carbamate **7**, a synthesis route via a carbamoyl imidazolium salt was established (see Scheme 2). Carbamoyl imidazolium salts are very stable and can easily be synthesized from carbonyldiimidazole [13,14,15] as shown in the synthesis scheme.

### 2.2. In Vitro Testing

The newly synthesized carbamates **2a**–**2d** and **7** were tested for their ability to inhibit histone deacetylases in vitro using a homogenous and fluorescence-based biochemical assay developed in our group and SAHA as a reference inhibitor [16]. In order to investigate a potential subtype selectivity among different HDAC classes, we first tested them against the recombinant histone deacetylases HDAC1 (class I) and HDAC6 (class llb). As depicted in Table 1, all substances inhibit the activity of both of these HDAC subtypes. Of all tested compounds, the water-soluble substance bearing a protonated piperazine residue (**7**) exhibits the weakest inhibitory activity with IC_50_ values of 5.1 µM and 3.7 µM, respectively. The least potent of the other carbamates is the one carrying an isopropyl residue (**2a**) with IC_50_ values in the low micromolar range, and all other carbamates **2b**–**2d** show HDAC inhibition in the nanomolar range with compound **2d**, holding a phenyl residue, being the most potent one with IC_50_ values of 132 nM and 187 nM. Although we tested only this small subset of substances, an initial structure-activity-relationship model can be set up by ranking the substitution patterns according to their inhibition properties. The observed inhibition of the HDAC subtypes 1 and 6 decreases in the following order: phenyl > benzyl, ethyl > isopropyl > piperazine. These test results clearly show that the different substitution patterns have a different impact on either the intrinsic enzyme inhibition of the carbamates or on their degradation and thus on the release of SAHA, respectively both. To further address this issue, we also tested the respective compounds against the class l histone deacetylase HDAC8 (also see Table 1). As expected, SAHA is much less potent against HDAC8 (IC_50_ = 5.7 µM) compared to the other tested subtypes. However, remarkably, only the phenyl compound **2d** is able to inhibit HDAC8 in a similar range (6.5 µM). All other carbamates show only weak inhibition at a concentration of 100 µM.

Importantly, the overall in vitro test results suggest an intrinsic inhibition of the carbamates themselves. All tested compounds inhibit HDAC1 and HDAC6, but the carbamates **2a**–**2c** showed little inhibition against HDAC8 under the same assay conditions. If the recorded inhibitory effects were completely a result of the degradation and release of SAHA, a stronger inhibition of HDAC8 should have been observed for these compounds, as well. In the case of **2d**, we observed only about a 5% release of SAHA, but basically the same inhibitory potency, which also implies a direct inhibition by the carbamate.

### 2.3. Stability Analysis

In order to address the question if the newly-synthesized carbamates are hydrolyzed during the assay procedure or if they really serve as HDAC inhibitors themselves, stability stress tests were performed. For this purpose, the compounds were subjected to the standard in vitro assay conditions, but without adding a substrate as, no enzyme conversion should be observed in these experiments. After 90 min of incubation at 37 °C, the release of SAHA of the different samples was detected and quantified via HPLC. This led to the finding that a release of SAHA could be detected for all samples (see Table 1). Notably, for the compounds **2a**–**2d**, SAHA was the only observed degradation product, while for compound **7**, several non-identified degradation products could be detected. The stability results show that variation of the substitution pattern indeed leads to a different degree of degradation increasing in the following order: benzyl < isopropyl < piperazine < phenyl < ethyl. However, remarkably, the degree of degradation does not correlate with the in vitro assay results at all. This means that the question if the carbamate scaffold represents a new warhead for HDAC inhibition or if the degradation to SAHA is solely responsible for the observed inhibitory effects cannot be resolved completely. However, the results strongly suggest that at least some of the tested carbamates are not just prodrugs, but HDAC inhibitors themselves.

### 2.4. Docking Studies

**Figure 1:**
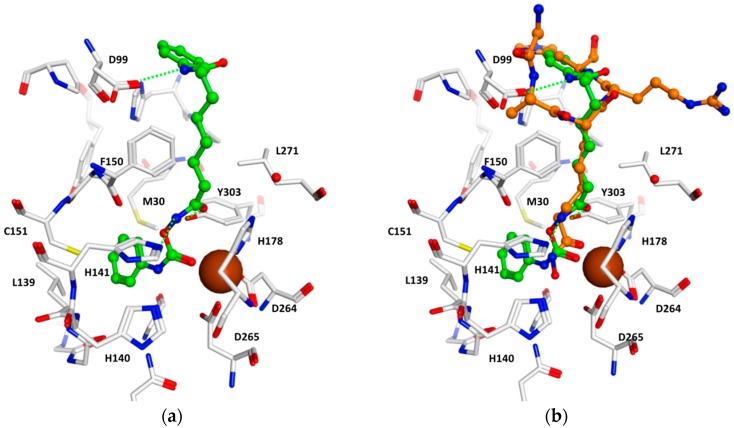
Docking results for HDAC1. (**a**) Interaction of **2d** (green) with HDAC1; (**b**) comparison of the docking pose for **2d** and the crystal structure of HDAC1 in complex with a hydroxamate peptide derived inhibitor (orange). The zinc ion is shown as a brown ball, and hydrogen bonds are drawn as dashed green lines.

To rationalize the obtained biochemical data, notably to analyze the putative binding mode, the synthesized inhibitors were docked to the available crystal structures of HDAC1 and HDAC6, which were recently solved in complex with inhibitors [17,18,19]. The docking results of the carbamates showed that these inhibitors are able to coordinate to the zinc ion by a carbonyl group of the carbamate. The terminal hydrophobic group is accommodated in the so-called foot pocket found in several HDAC isoforms. In HDAC1, this pocket is formed by several hydrophobic residues (Met30, Leu139 and Cys151, see Figure 1). The aromatic phenyl ring of the carbamate **2d** fits perfectly into this foot pocket (see Figure 1a). The foot pocket was found in crystal structures of the related HDAC2 to be the binding site for the aromatic ring of anilinobenzamide derived inhibitors [20]. Two further hydrogen bonds are observed between the hydroxamate carbamate group and His141/Tyr303. In addition, the SAHA-like inhibitors **2a**–**2d** interact with the conserved phenyl residues of the acetyl-lysine tunnel and make a hydrogen bond with Asp99 at the rim of the pocket as observed for the cocrystallized peptidic inhibitor (see Figure 1b).

**Figure 2:**
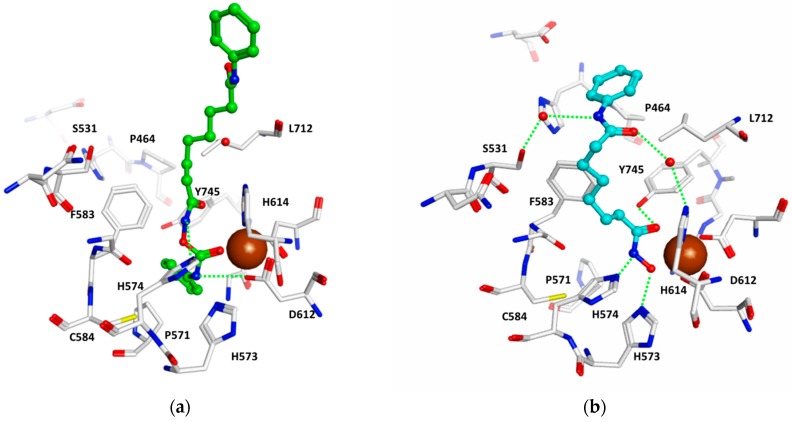
Docking results for HDAC6. (**a**) Interaction of **2d** (green) with HDAC6; (**b**) crystal structure of HDAC6 in complex with the inhibitor SAHA (cyan, PDB ID 5GOI). The zinc ion is shown as a brown ball, and hydrogen bonds are drawn as dashed green lines.

**Figure 3:**
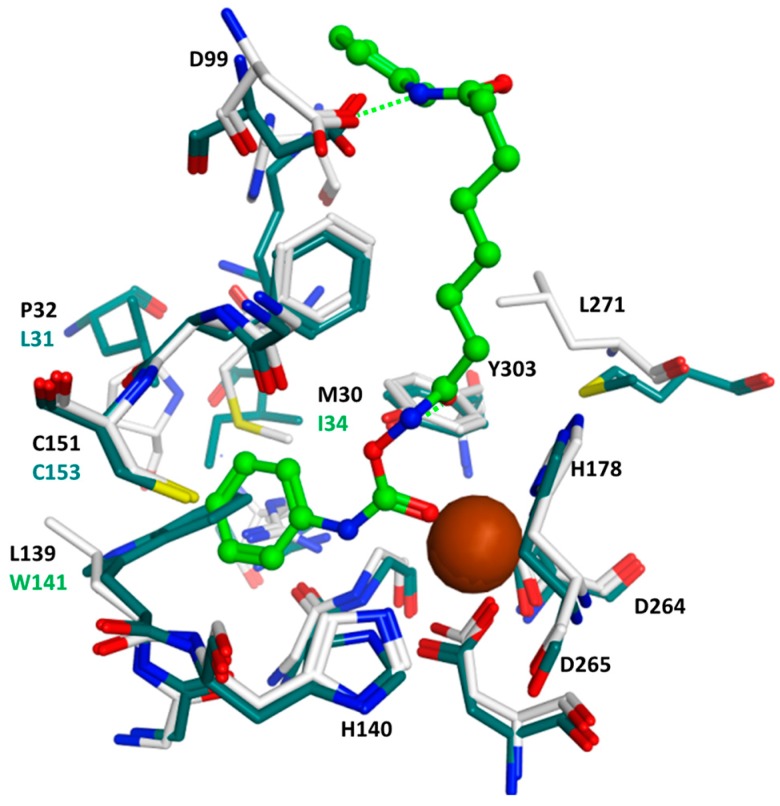
Comparison of the HDAC1-**2d** docking complex (same colors as in Figure 2) and the HDAC8 crystal structure (PDB ID 2V5X, colored dark green). The residues of HDAC8 forming the foot pocket are labeled dark green.

A similar orientation was observed in the case of HDAC6 (see Figure 2). Furthermore, here, the terminal aliphatic/aromatic substituent is located in the foot pocket, whereas the carbamate coordinates to the zinc ion and makes two hydrogen bonds to His574/Asp612 (see Figure 2a). However, a plausible binding mode was only derived when Tyr745 was considered in a moved-out conformation as observed in the crystal structure of HDAC8 in complex with an amino acid-derived inhibitor addressing the foot pocket, whereas the classical hydroxamate-derived inhibitors prefer a moved-in conformation, which enables a further hydrogen bond between the conserved tyrosine and the hydroxamate carbonyl group (see Figure 2b). In the case of HDAC8, the foot pocket is restricted by the bulky Trp141, and the inhibitors are not able to interact with the Zn-ion as observed in HDAC1 and 6 (see Figure 3).

### 2.5. Cellular Testing

To further investigate the inhibition properties of the carbamates, we tested their antiproliferative activity on human promyelocytic leukemia cells (HL60) and on human histiocytic lymphoma cells (U937). As depicted on the left side of Table 2, all compounds besides the piperazine derivative block proliferation of the tested cell types in the low micromolar range, with carbamate **2d**, which is substituted with a phenyl ring, showing antiproliferative effects in the same range as SAHA.

**Figure 4:**
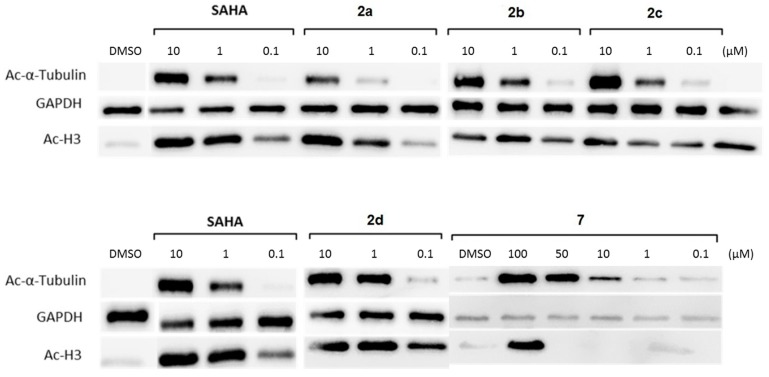
Detection of acetylation levels of H3 and tubulin after incubation of HL60 cells with SAHA and the carbamates **2a**–**2d** and **7**. GAPDH was used as a loading control.

In order to show that the inhibition of proliferation in fact is based on HDAC inhibition on a cellular level, the effect of the inhibitors on acetylation levels of HDAC target proteins in HL60 cells was tested via Western blot. An inhibition of deacetylation in this experiment is expected to lead to hyperacetylation of the respective proteins. Besides histone H3, acetylated tubulin was chosen as a target protein. As tubulin is only accepted as a target by HDAC 6 [21], this allows us to explore subtype selectivity in a cellular setting (see Figure 4).

The results mirror the antiproliferative effects that were shown above. For instance, Compound **2d** is affecting H3 and tubulin acetylation levels in the same range as SAHA, and Compound **7** was the least effective substance. As expected from the in vitro tests, none of the inhibitors shows selectivity for tubulin or H3 hyperacetylation. It could be shown that the substrate used in our biochemical in vitro assay is cell permeable and converted by HDACs within living cells [22,23]. Therefore, we decided to adapt this assay for a cellular setting allowing us to verify the previous results by assessing HDAC inhibition in the human leukemia cell lines HL60 and U937 (see the right side of Table 2). Remarkably, all described cellular tests clearly demonstrate that our carbamates have effects on HDAC inhibition at the cellular level. It can be assumed that the differences in inhibition of proliferation and cellular HDAC inhibition are the result of varying cell permeability or solubility due to the different substitution patterns. However, of course, it cannot be completely excluded that a different degree of degradation to SAHA is partially responsible for the different test results.

### 2.6. Carbamate of Bufexamac

**Scheme 3:**
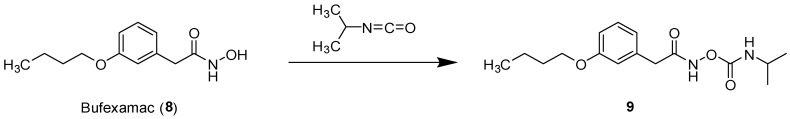
Route of synthesis for the carbamate derived from bufexamac. *Reaction conditions*: dimethylformamide, acetone, −15 °C.

To further elucidate the question, if the carbamates show an intrinsic HDAC inhibition, we decided to prepare a carbamate of the known HDAC6-selective inhibitor bufexamac (**8**; see Scheme 3) and explore this derivative’s inhibition properties.

Notably, a change in subtype selectivity compared to the parent compound would also underline that carbamates in fact are able to inhibit HDACs without being degraded. After testing both bufexamac and the carbamate **9** against the two histone deacetylase subtypes HDAC1 and HDAC6, it became obvious that the carbamate **9** even shows improved selectivity for HDAC6 over HDAC1 compared to bufexamac (see Table 3). This effect is clearly not explainable by the degradation of the compound under the release of hydroxamic acid, strongly implying that the carbamate function in **9** is contributing to the HDAC inhibition to a great extent.

**Figure 5:**
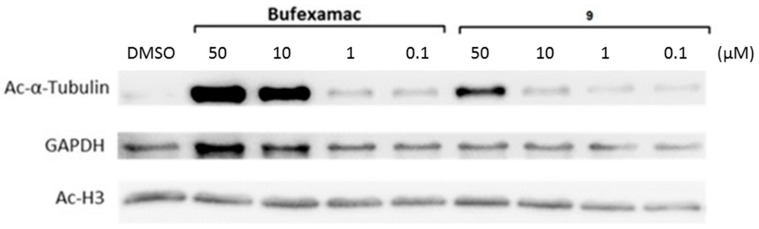
Detection of acetylation levels of H3 and tubulin after incubation of HL60 cells with bufexamac and the carbamate 9. GAPDH was used as a loading control.

To verify this finding on a cellular level, we also assessed the antiproliferative properties and the effects on target protein acetylation of the carbamate **9** and of bufexamac. Table 3 also contains the GI_50_ values of these two inhibitors demonstrating that both compounds lead to comparable block of proliferation in HL60 cells. Figure 5 shows the results of Western blot experiments in the same cell line. Again, H3 and the HDAC6 substrate tubulin were chosen as target proteins, allowing us to test substrate selectivity at the cellular level. As both tested compounds lead to a hyperacetylation of tubulin, while the acetylation levels of H3 are not affected, this target validation experiment also shows that both the carbamate **9** and its parent substance bufexamac possess subtype selectivity towards HDAC6 over HDAC1.

To test the stability of Compound **9** under assay conditions, we also performed stability analysis with HPLC quantification. This experiment led to the finding that 8.7% of the carbamate **9** is degraded under release of bufexamac during incubation time. This fraction of released bufexamac may contribute to in vitro and cellular HDAC inhibition, but the observed selectivity profile of the carbamate in the in vitro assays clearly implies an intrinsic HDAC activity of carbamates.

## 3. Materials and Methods

### 3.1. Synthesis

#### 3.1.1. General

The starting materials (chemicals) were purchased from Acros Organics, Sigma-Aldrich, Alfa Aesar and Fluka and were used in synthesis without any further purification. Analytical thin-layer chromatography (TLC) was performed with TLC Silica gel 60 F254 plates from Merck. All yields were unoptimized. Nuclear magnetic resonance (NMR) spectroscopy was performed on an Avance DRX Spectrometer (^1^H NMR: 400 Hz, ^13^C NMR: 100 Hz) from Bruker. Chemical shifts were reported in parts per million (ppm, δ) downfield from tetramethylsilane, and compounds were dissolved in CDCl_3_ or DMSO-*d*_6_. Proton coupling patterns were described as singlet (s), broad singlet (bs), doublet (d), doublet of doublets (dd), triplet (t) and multiplet (m). Mass spectra with electrospray ionization (ESI) were recorded on a LCQ Advantage spectrometer (Thermo). For all tested compounds, a purity of >95% was determined by HPLC and UV detection (λ = 210 nm). HPLC analyses were performed on an Alliance 2695 Separation Module (Waters) or on an Agilent 1200 Series device (Agilent, Santa Clara, CA, USA) with these analytical columns: LiChrospher^®^ RP-select B, 5 µm, 60 Å, 250 × 4 mm (Merck); Kinetex. 5u XB-C18, 100 Å, 250 × 4.6 mm (Phenomenex, Torrance, CA, USA); using the following conditions: Eluent A: H_2_O containing 0.05% TFA, Eluent B: acetonitrile containing 0.05% TFA, linear gradient conditions (0–4 min, A = 90%, B = 10%; 4–29 min, linear increase to 100% of B; 29–31 min, B = 100%; 31.5–40 min, A = 90%, B = 10%). For evaluation, the ChemStation software from Agilent was used.

#### 3.1.2. General Procedure for the Preparation of the Carbamates **2a**–**2d** and **9**

A solution of 1.1 eq SAHA (synthesized as described in the literature [10,11] or bufexamac (purchased from Sigma Aldrich, St. Louis, MO, USA) in DMF (1 mL/mmol) and acetone (2 mL/mmol) was cooled to −15 °C. After 1 eq of the substituted isocyanate was slowly added, the batch was stirred over night at room temperature. The solvent was removed under reduced pressure, and the desired carbamate was precipitated by the addition of water. The raw product was purified by recrystallization from ethyl acetate.

*[(8-Anilino-8-oxo-octanoyl)amino]N-isopropylcarbamate* (**2a**). Remained as a white powder (0.324 g, 93% yield); ^1^H NMR (400 MHz, DMSO-*d*_6_): δ (ppm) = 11.24 (s, 1H, NH), 9.84 (s, 1H, NH), 7.61–7.54 (m, 3H, NH, Ar2,6-H), 7.31–7.25 (m, 2H, Ar3,5-H), 7.04–6.99 (m, 1H, Ar4-H), 3.64–3.53 (m, 1H, CH-(CH_3_)_2_), 2.29 (t, ^3^*J* = 7.4, 2H, Ar-NH-CO-CH_2_), 2.06 (t, ^3^*J* = 7.9, 2H, NH-CO-CH_2_), 1.63–1.48 (m, 4H, (CH_2_)_2_), 1.34–1.27 (m, 4H, (CH_2_)_2_), 1.08 (d, ^3^*J* = 6.9, 6H, CH-(CH_3_)_2_); ^13^C NMR (100 MHz, DMSO-*d*_6_): δ (ppm) = 171.62 (CO), 170.42 (CO), 154.51 (COO), 139.77 (Ar-C1), 129.04 (Ar-C3,5), 123.33 (Ar-C4), 119.42 (Ar-C2,6), 43.46 (CH(CH_3_)_2_), 36.80 (Ar-NH-CO-CH_2_), 32.36 (NH-CO-CH_2_), 28.80 (CH_2_), 28.68 (CH_2_), 25.42 (CH_2_), 25.09 (CH_2_), 22.80 ((CH_3_)_2_); MS-ESI^+^: *m*/*z* 350 [M + H]^+^, 372 [M + Na]^+^.

*[(8-Anilino-8-oxo-octanoyl)amino]N-benzylcarbamate* (**2b**). Remained as a white solid (0.373 g, 94% yield); ^1^H NMR (400 MHz, DMSO-*d*_6_): δ (ppm) = 11.36 (s, 1H, NH), 9.84 (s, 1H, NH), 8.22 (t, ^3^*J* = 5.8, 1H, NH-CH_2_), 7.60–7.56 (m, 2H, NH-Ar2,6-H), 7.36–7.22 (m, 7H, NH-Ar3,5-H, CH_2_-Ar2,3,4,5,6-H), 7.04–6.99 (m, 1H, NH-Ar4-H), 4.23 (d, ^3^*J* = 6.0, 2H, CH_2_-Ar), 2.28 (t, ^3^*J* = 7.4, 2H, Ar-NH-CO-CH_2_), 2.07 (t, ^3^*J* = 7.9, 2H, NH-CO-CH_2_), 1.63–1.47 (m, 4H, (CH_2_)_2_), 1.36–1.24 (m, 4H, (CH_2_)_2_); ^13^C NMR (100 MHz, DMSO-*d*_6_): δ (ppm) = 171.63 (CO), 170.54 (CO), 155.79 (COO), 139.75 (Ar-C1), 139.49 (Ar-C1), 129.04 (CH_2_-Ar-C2,6), 128.72 (CH_2_-Ar-C3,5), 127.46 (NH-Ar-C3,5), 127.38 (CH_2_-Ar-C4), 123.32 (NH-Ar-C4), 119.43 (NH-Ar-C2,6), 44.49 (CH_2_-Ar), 36.78 (Ar-NH-CO-CH_2_), 32.35 (NH-CO-CH_2_), 28.81 (CH_2_), 28.67 (CH_2_), 25.41 (CH_2_), 25.09 (CH_2_); MS-ESI^+^: *m*/*z* 398 [M + H]^+^, 420 [M + Na]^+^.

*[(8-Anilino-8-oxo-octanoyl)amino]N-ethylcarbamate* (**2c**). Remained as a white powder (0.310 g, 92% yield); ^1^H NMR (400 MHz, DMSO-*d*_6_): δ (ppm) = 11.27 (s, 1H, NH), 9.84 (s, 1H, NH), 7.65–7.55 (m, 3H, NH, Ar2,6-H), 7.31–7.25 (m, 2H, Ar3,5-H), 7.04–6.99 (m, 1H, Ar4-H), 3.09–2.99 (m, 2H, CH_2_-CH_3_), 2.29 (t, ^3^*J* = 7.3, 2H, Ar-NH-CO-CH_2_), 2.07 (t, ^3^*J* = 7.9, 2H, NH-CO-CH_2_), 1.62–1.46 (m, 4H, (CH_2_)_2_), 1.35–1.24 (m, 4H, (CH_2_)_2_), 1.03 (t, ^3^*J* = 7.2, 3H, CH_2_-CH_3_); ^13^C NMR (100 MHz, DMSO-*d*_6_): δ (ppm) = 171.69 (CO), 170.42 (CO), 155.20 (COO), 139.82 (Ar-C1), 129.04 (Ar-C3,5), 123.33 (Ar-C4), 119.43 (Ar-C2,6), 36.76 (Ar-NH-CO-CH_2_), 35.90 (CH_2_-CH_3_), 32.35 (NH-CO-CH_2_), 28.79 (CH_2_), 28.67 (CH_2_), 25.42 (CH_2_), 25.12 (CH_2_), 14.26 (CH_3_); MS-ESI^+^: *m*/*z* 336 [M + H]^+^, 358 [M + Na]^+^.

*[(8-Anilino-8-oxo-octanoyl)amino]N-phenylcarbamate* (**2d**). Remained as a white solid (0.368 g, 96% yield); ^1^H NMR (400 MHz, DMSO-*d*_6_): δ (ppm) = 11.54 (s, 1H, NH), 10.15 (s, 1H, NH), 9.82 (s, 1H, NH), 7.61–7.55 (m, 2H, Ar2,6-H), 7.48–7.43 (m, 2H, Ar2′,6′-H), 7.35–7.23 (m, 4H, Ar3,3′,5,5′-H) 7.08–6.98 (m, 2H, Ar4,4′-H), 2.29 (t, ^3^*J* = 7.2, 2H, Ar-NH-CO-CH_2_), 2.14 (t, ^3^*J* = 7.2, 2H, NH-CO-CH_2_), 1.65–1.48 (m, 4H, (CH_2_)_2_), 1.39–1.24 (m, 4H, (CH_2_)_2_); ^13^C NMR (100 MHz, DMSO-*d*_6_): δ (ppm) = 171.62 (CO), 170.69 (CO), 152.74 (COO), 139.77 (Ar-C1), 138.68 (Ar-C1′), 129.35 (Ar-C3,5), 129.02 (CH_2_-Ar-C3′,5′), 123.50 (Ar-C4), 123.31 (Ar-C4′), 119.48 (Ar-C2,6), 118.82 (Ar-C2′,6′), 36.81 (Ar-NH-CO-CH_2_), 32.82 (NH-CO-CH_2_), 28.82 (CH_2_), 28.57 (CH_2_), 25.41 (CH_2_), 25.08 (CH_2_); MS-ESI^+^: *m*/*z* 384 [M + H]^+^, 406 [M + Na]^+^.

*[[2-(4-Pentoxyphenyl)acetyl]amino]N-isopropylcarbamate* (**9**). Remained as a white solid (0.272 g, 88% yield); ^1^H NMR (400 MHz, DMSO-*d*_6_): δ (ppm) = 11.49 (s, 1H, NH), 7.60 (d, ^3^*J* = 3.16, 1H, NH), 7.17 (d, ^3^*J* = 3.7, 2H, Ar3,5-H), 6.85 (d, ^3^*J* = 4.2, 2H, Ar2,6-H), 3.93 (t, ^3^*J* = 6.3, 2H, CH_2_-O), 3.64–3.53 (m, 1H, CH(CH3)_2_), 1.73–1.63 (m, 2H, CH_2_), 1.48–1.37 (m, 2H, CH_2_), 1.08 (d, ^3^*J* = 3.7, 6H, C(CH_3_)_2_), 0.93 (t, ^3^*J* = 7.4, 3H, CH_2_-CH_3_); remark: the two benzylic protons overlap with the signal of the solvent, but could be identified and assigned in a two-dimensional NMR experiment; ^13^C NMR (100 MHz, DMSO-*d*_6_): δ (ppm) = 168.36 (CO), 157.90 (COO), 154.41 (Ar-C4), 130.46 (Ar-C3,5), 127.29 (Ar-C1), 114.61 (Ar-C2,6), 67.52 (CH_2_-O), 43.42 (CH(CH_3_)_2_), 38.34 (Ar-CH_2_), 31.21 (CH_2_), 22.81 (CH_2_), 19.16 (CH_3_), 14.08 (C(CH_3_)_2_); MS-ESI^+^: *m*/*z* 398.0 [M + H]^+^, 420.0 [M + Na]^+^.

*tert-Butyl-piperazine-1carboxylate* (**3**). A solution of 5 mmol di-*tert*-butyl dicarbonate in dichloromethane (5 mL/mmol) was slowly added to a solution of piperazine (10 mmol) in dichloromethane (25 mL/mmol). The batch was stirred over night before the solvent was removed under reduced pressure. The addition of water led to precipitation of the raw product, which was filtrated and extracted with dichloromethane. The combined organic phases were dried over sodium sulfate and evaporated to result in 0.616 g of white solid (66% yield). ^1^H NMR (400 MHz, CDCl_3_): δ (ppm) = 3.40 (t, ^3^*J* = 5.0, 4H, N-(CH_2_)_2_), 2.82 (t, ^3^*J* = 5.2, 4H, HN-(CH_2_)_2_), 1.47 (s, 9H, C(CH3)3); ^13^C NMR (100 MHz, CDCl_3_): δ (ppm) = 154.88 (CO), 79.41 (C(CH_3_)_3_), 45.88 (N-(CH_2_)_2_), 44.66 (N-(CH_2_)_2_), 28.38 (C(CH_3_)_3_).

*tert-Butyl 4-(imidazole-1-carbonyl)piperazine-1-carboxylate* (**4**). Three-point-three millimoles *tert*-butyl-piperazine-1carboxylate (3) were dissolved in dichloromethane (5 mL/mmol) and added dropwise to a solution of carbonyldiimidazole (CDI, 3.63 mmol) in dichloromethane (5 mL/mmol) under ice cooling. The batch was stirred over night at room temperature before the solvent was removed under reduced pressure, and the raw product was purified via flash chromatography resulting in 0.850 g of a white solid (91% yield). ^1^H NMR (400 MHz, CDCl_3_): δ (ppm) = 7.91–7.90 (m, 1H, imidazole2-H), 7.22–7.21 (m, 1H, imidazole4-H), 7.13–7.12 (m, 1H, imidazole5-H), 3.62–3.58 (m, 4H, N-(CH_2_)_2_), 3.56–3.52 (m, 4H, N-(CH_2_)_2_), 1.49 (s, 9H, C(CH_3_)_3_); ^13^C NMR (100 MHz, CDCl_3_): δ (ppm) = 154.25 (CO), 150.92 (CO), 136.81 (imidazole-C2), 130.01 (imidazole-C4), 117.77 (imidazole-C5), 80.72 (C(CH_3_)_3_), 46.26 (N-(CH_2_)_2_), 43.18 (N-(CH_2_)_2_), 28.19 (C(CH_3_)_3_).

*tert-Butyl 4-(3-methylimidazol-3-ium-1-carbonyl)piperazine-1-carboxylate iodide* (**5**). Three millimoles of the carbamoyl imidazole (4) were dissolved in acetonitrile (5 mL/mmol), and 16 mmol methyl iodide were slowly added dropwise under ice cooling. The batch was stirred over night at room temperature. The precipitate was filtrated and washed with diethyl ether resulting in 0.760 g of a light yellow solid (60% yield). ^1^H NMR (400 MHz, DMSO-*d*_6_): δ (ppm) = 9.56–9.55 (m, 1H, Imidazole2-H), 7.86–7.84 (m, 1H, Imidazole4-H), 7.64–7.63 (m, 1H, Imidazole5-H), 3.86 (s, 1H, N-CH_3_), 3.50–3.40 (m, 8H, N-(CH_2_)_2_-(CH_2_)_2_), 1.37 (s, 9H, C(CH_3_)_3_); ^13^C NMR (100 MHz, DMSO-*d*_6_): δ (ppm) = 154.57 (CO), 147.27 (CO), 137.44 (Imidazole-C2), 123.96 (Imidazole-C4), 121.27 (Imidazole-C5), 80.68 (C(CH_3_)_3_), 46.11 (N-(CH_2_)_2_), 42.62 (N-(CH_2_)_2_), 36.76 (N-CH_3_), 28.35 (C(CH_3_)_3_).

*O4-[(8-Anilino-8-oxo-octanoyl)amino]O1-tert-butyl piperazine-1,4-dicarboxylate* (**6**). One-point-six-three millimoles of the carbamoyl imidazolium salt (**5**) were dissolved in acetone and added dropwise to a solution of 1.5 mmol SAHA (1) in DMF (1 mL/mmol). After stirring over night at room temperature, the solvent was removed under reduced pressure, and water was added to the remaining solution. The raw product was extracted with dichloromethane, and the combined organic phases were dried over sodium sulfate. After removal of the solvent, the product was purified via flash chromatography (ethyl acetate/cyclohexane 9:1) to result in 0.240 g of a white solid (34% yield). ^1^H NMR (400 MHz, CDCl_3_): δ (ppm) = 9.29 (s, 1H, NH), 7.78 (s, 1H, NH), 7.57–7.51 (m, 2H, Ar2,6-H), 7.34–7.29 (m, 2H, Ar3,5-H), 7.13–7.07 (m, 1H, Ar4-H), 3.57–3.41 (m, 8H, N-(CH_2_)_2_-(CH_2_)_2_), 2.37 (t, ^3^*J* = 7.4, 2H, Ar-NH-CO-CH_2_), 2.25 (t, ^3^*J* = 7.4, 2H, NH-CO-CH_2_), 1.79–1.66 (m, 4H, (CH_2_)_2_), 1.48 (s, 9H, C(CH_3_)_3_), 1.43–1.39 (m, 4H, (CH_2_)_2_). ^13^C NMR (100 MHz, CDCl_3_): δ (ppm) = 171.98 (CO), 171.72 (CO), 154.42 (COO), 154.11 (CO), 138.08 (Ar-C1), 128.91 (Ar-C3,5), 124.08 (Ar-C4), 119.74 (Ar-C2,6), 80.43 (C-CH_3_), 44.17 (N-(CH_2_)_2_), 43.10 (N-(CH_2_)_2_), 37.03 (Ar-NH-CO-CH_2_), 32.49 (NH-CO-CH_2_), 28.31 (C(CH_3_)_3_), 28.12 (CH_2_), 27.95 (CH_2_), 25.04 (CH_2_), 24.59 (CH_2_). MS-ESI^+^: *m*/*z* 477.2 [M + H]^+^, 499.3 [M + Na]^+^.

*[(8-Anilino-8-oxo-octanoyl)amino]piperazin-4-ium-1-carboxylate trifluoroacetate* (**7**). Zero-point-one millimoles of the Boc-protected amine (6) were dissolved in dichloromethane (5 mL/mmol) and cooled to 0 °C. Trifluoroacetic acid (3 mL/mmol) was slowly added dropwise. The batch was stirred for 2 h at room temperature, and the reaction progress periodically was detected via thin layer chromatography. After complete elimination of the protecting group was detected, the solvent was removed under reduced pressure. This was done repetitively until the typical scent of trifluoroacetic acid was no longer recognized. After the final evaporation step, 0.043 g of a white solid remained (90% yield). ^1^H NMR (400 MHz, DMSO-*d*_6_): δ (ppm) = 11.62 (s, 1H, NH), 9.86 (s, 1H, NH), 9.06 (bs, 2H, NH_2_), 7.61–7.56 (m, 2H, Ar2,6-H), 7.31–7.24 (m, 2H, Ar3,5-H), 7.04–6.99 (m, 1H, Ar4-H), 3.62 (bs, 4H, N-(CH_2_)_2_, 3.14 (t, ^3^*J* = 5.0, 4H, (CH_2_)_2_-NH_2_), 2.29 (t, ^3^*J* = 7.3, 2H, Ar-NH-COCH_2_), 2.10 (t, ^3^*J* = 7.2, 2H, NH-CO-CH_2_), 1.61–1.48 (m, 4H, (CH_2_)_2_), 1.33–1.27 (m, 4H, (CH_2_)_2_). ^13^C NMR (100 MHz, DMSO-*d*_6_): δ (ppm) = 171.34 (CO), 170.58 (CO), 158.73 (q, ^2^JC-C-F = 33, CO-CF_3_), 153.74 (COO), 139.75 (Ar-C1), 129.02 (Ar-C3,5), 123.34 (Ar-C4), 119.41 (Ar-C2,6), 42.79 (N-(CH_2_)_2_), 41.39 (N-(CH_2_)_2_), 36.76 (Ar-NH-CO-CH_2_), 32.18 (NH-CO-CH_2_), 28.78 (CH_2_), 28.65 (CH_2_), 25.38 (CH_2_), 25.07 (CH_2_). MS-ESI^+^: *m*/*z* 377 [M + H]^+^.

### 3.2. Enzymes and In Vitro Testing

Recombinant human HDAC1 and 6 were purchased from BPS Biosciences (San Diego, CA, USA), and recombinant human HDAC8 was produced as described before [24]. Inhibition tests of human HDAC1 and 6 were conducted using carboxybenzyl acetyllysine aminomethylcoumarin (ZMAL) as a substrate. After incubation of the test compound, ZMAL (10.5 μM) and enzyme, for 90 min at 37 °C, 60 μL of a trypsin solution, were added, and the mixture was further incubated for 20 min at 37 °C. For testing of HDAC8, the commercial Fluor de Lys drug discovery kit (BML-KI178) (Enzo Life Sciences, Lausen, Switzerland) was used according to the manufacturer’s instruction. Briefly, the test compound, Fluor de Lys-HDAC8 substrate (50 μM), and enzyme were incubated for 90 min at 37 °C with subsequent addition of 50 μL of Developer II (BML-KI176) (Enzo Life Sciences, Lausen, Switzerland) and further incubation for 45 min at 30 °C. In both assays, trichostatin A (2 μM) was used to stop the reaction. Fluorescence in both cases was measured in a plate reader (BMG Polarstar, BMG Labtech, Ortenberg, Germany) with excitation at λ = 390 nm and emission at λ = 460 nm. IC_50_ values were determined with GraphPad Prism 4.02 (GraphPad Software, La Jolla, CA, USA).

### 3.3. Docking Studies

Crystal structures of human HDAC1 (PDB ID 5ICN) [17] and human HDAC6 catalytic domain 2 (PDB IDs 5EEN, 5EF8, 5GOI) [18,19] were used for the docking study and were downloaded from the Protein Data Bank (PDB) [25]. Protein preparation was done using Schrödinger’s Protein Preparation Wizard [26] by adding hydrogen atoms, assigning protonation states and minimizing the protein structure. Ligands were prepared in MOE (Molecular Operating Environment) [27] from smiles in neutral form. Multiple low energy starting conformations were generated with MOE within an energy window of 5 kcal/mol. Molecular docking was performed using program Glide software [26]. The same protocol was used as in previous studies [28,29]. A conserved water molecule bound to His178 (HDAC1) or His614 (HDAC6) was included. In the case of HDAC6, Tyr745 was considered to be flexible, and two different side chain conformations were considered as observed in the crystal structures of HDAC8 in complex with inhibitors addressing the foot pocket [30]. The best docking pose was selected based on the Glide score. The docking protocol was tested using the cocrystallized inhibitors in HDAC1 and HDAC6 (PDB IDs 5ICN, 5EEN, 5EF8, 5GOI). The docking protocol was able to reproduce the experimentally-derived binding mode of the inhibitors with RMSD values below 1.2 Å.

### 3.4. Cellular Testing

#### 3.4.1. Cell Proliferation Assay

HL60 or U937 cells were seeded in 96-well tissue culture plates at a density of 5 × 10^4^ cells per well. Cells were immediately incubated with inhibitors. As these cells already differentiate with 1% DMSO content, inhibitors were prepared as 200× stock solutions in DMSO. In all cases, inhibitors were compared to DMSO vehicle only, and three replicates per concentration were used. Growth inhibition was determined using the CellTiter 96^®^ AQueous Non-Radioactive Cell Proliferation Assay according to the manufacturer’s instructions. Briefly, 5 × 10^4^ of HL60 or U937 cells per well were treated with DMSO stocks of the respective inhibitors or with DMSO in 100 µL cell culture medium and incubated for 72 h at 37 °C and 5% CO_2_ in transparent 96-well microtiter plates (PAA). Subsequently, 20 µL of the detection reagent (containing a tetrazolium salt) was added to each well and incubated for 4 more hours under the same conditions. After this time, absorption was measured at a wavelength of 490 nm (BMG Polarstar, BMG Labtech, Ortenberg, Germany). Data were plotted as absorbance units against the logarithm of compound concentration using GraphPad Prism 4.02 (GraphPad Software, La Jolla, CA, USA). Fifty percent growth inhibition (GI_50_) was determined as the compound concentration required to reduce the number of metabolically-active cells by 50% compared to DMSO control.

#### 3.4.2. Acetylation Levels

HL60 cells were treated with the inhibitors in the given concentrations. Cells treated with dimethyl sulfoxide as a vehicle were used as controls. After an incubation time of 4 h, cells were transferred into 50-mL Falcon tubes and centrifuged (300× *g*, 5 min). The supernatant was removed, and the cells were washed with PBS, before they were transferred to 1.7-mL reaction tubes. The cells were lysated with Bio-Rad loading buffer, and the cell suspension was heated to 95 °C for 5 min. The proteins then were separated using a 15% SDS-polyacrylamide gel electrophoresis and transferred to a polyvinylidene fluoride (PVDF) membrane (Roti-PVDF, Carl Roth, Karlsruhe, Germany). Acetylation levels were detected using an anti-acetyl histone H3 antibody (Millipore, Billerica, MA, USA) and an anti-acetyl-α-tubulin antibody (Sigma Aldrich, St. Louis, MO, USA). An antibody directed against GAPDH (Sigma Aldrich) was used as a loading control.

#### 3.4.3. Cellular HDAC Inhibition Assay

To assess cellular inhibition of HDAC activity, 7.5 × 10^4^ cells per well in 49.75 µL media were treated with 0.25 µL test compound or DMSO as a control at 37 °C and 5% CO_2_. After 2 h of incubation, 50 µL substrate solution were added, containing *tert*-butyloxycarbonyl acetyl lysine aminomethylcoumarin (MAL) as a substrate (300 µM final assay concentration) and IGEPAL CA-630 (final concentration 0.05%). After 2 more hours of incubation at 37 °C and 5% CO_2_, 50 µL stop solution consisting of 0.5 µL TSA (100 µM) and 5 µL trypsin solution (10 mg/mL) in 44.5 µL cell buffer were added. This mixture was incubated for a further 20 min at 37 °C. Fluorescence was measured in a plate reader (BMG Polarstar) with excitation at λ = 390 nm and emission at λ = 460 nm. IC_50_ values were determined with GraphPad Prism 4.02.

### 3.5. Stability Analysis

For stability tests, the carbamates were subjected to the described in vitro assay conditions (see above) without adding the substrate ZMAL and without the second incubation step with trypsin. Compounds were applied in a final concentration of 2.4 mM, and recombinant HDAC1 was used as the enzyme. After 90 min of incubation at 37 °C, the release of SAHA was detected via HPLC on an Agilent 1200 series device. The employed analytical column was a Kinetex 5u XB-C18, 100 Å, 250 × 4.6 mm (Phenomenex, Torrance, CA, USA) with the ReproSil 100 C18 5-µm precolumn (Jasco, Oklahoma City, OK, USA). The following conditions were applied: Eluent A: H_2_O containing 0.05% TFA, Eluent B: acetonitrile containing 0.05% TFA, linear gradient conditions (0–4 min, A = 90%, B = 10%; 4–29 min, linear increase to 100% of B; 29–31 min, B = 100%; 31.5–40 min, A = 10%, B = 90%); injection volume: 30 µL, flow rate: 1 mL/min; detection wavelength: λ = 254 nm. For evaluation, the ChemStation software from Agilent was used. To quantify the released amount of SAHA and bufexamac, calibration curves were plotted by incubating DMSO stocks of these compounds under in vitro assay conditions with final compound concentrations ranging from 1 µM–200 µM. HPLC analysis with the conditions described above led to the following equations: *y* = 13.935*x* − 27.918 for SAHA and *y* = 0.6099*x* + 1.1301 for bufexamac.

## 4. Conclusions

The aim of this study was to validate our previously-postulated prodrug concept for hydroxamic acid containing HDAC inhibitors, which would be beneficial, e.g., for increased aqueous solubility or improved cellular uptake. However, the synthesis and evaluation of a subset of carbamates of the FDA-approved HDAC inhibitor SAHA strongly suggest that at least some of the synthesized carbamates are potent inhibitors themselves, representing a completely new warhead for HDAC inhibition. Generally, in light of the similar cellular potency of hydroxamates and some of the carbamates in the cells, we assume a weaker binding of the carbamate function to the zinc and/or steric hindrance for decreased in vitro potency, but an improved cellular uptake and possibly increased cleavage to the hydroxamate in the cellular setting. Thus, this study paves the way toward further analysis of carbamates of hydroxamates as new HDAC inhibitor warheads, but still shows potential for carbamates as prodrugs for hydroxamates.

To refine and validate these findings, a larger library of carbamates of different hydroxamic acid containing HDAC inhibitors should be set up and tested for HDAC inhibition, selectivity and stability.

## Figures and Tables

**Table 1:** In vitro test results of SAHA and the carbamates against the HDAC subtypes 1, 6 and 8. The table also contains the results of the stability tests under in vitro assay conditions (right column).

Compound	IC_50_ (µM) ± SE or Inhibition (%) @ Conc. (µM)			Release of SAHA (%)
	HDAC1	HDAC6	HDAC8	
**SAHA (1)**	0.065 ± 0.002	0.061 ± 0.002	5.74 ± 0.08	-
**2a**	1.52 ± 0.02	2.11 ± 0.02	35 @ 100	2.2
**2b**	0.74 ± 0.01	0.637 ± 0.002	52 @ 100	0.5
**2c**	0.79 ± 0.01	0.58 ± 0.01	68 @ 100	14.5
**2d**	0.132 ± 0.001	0.187 ± 0.007	6.49 ± 0.12	4.6
**7**	5.09 ± 0.26	3.71 ± 0.01	not tested	3.7

**Table 2:** Antiproliferative effects of SAHA and the carbamates **2a**–**2d** and 7 on HL60 and U937 cells (left side, GI_50_) and their HDAC inhibition in the cellular setting (right side, IC_50_).

Compound	GI_50_ (µM) ± SE		IC_50_ (µM) ± SE	
	HL60	U937	HL60	U937
**SAHA**	0.54 ± 0.03	0.43 ± 0.09	0.65 ± 0.05	0.73 ± 0.005
**2a**	1.02 ± 0.09	0.49 ± 0.09	3.25 ± 0.02	5.30 ± 0.59
**2b**	1.06 ± 0.11	0.68 ± 0.09	1.89 ± 0.04	3.02 ± 0.03
**2c**	0.99 ± 0.17	0.88 ± 0.13	1.42 ± 0.53	2.07 ± 0.04
**2d**	0.58 ± 0.05	0.42 ± 0.13	0.69 ± 0.004	0.71 ± 0.002
**7**	25.92 ± 1.96	not tested	22.35 ± 0.28	not tested

**Table 3:** In vitro test results of bufexamac and the carbamate **9** against the HDAC subtypes 1 and 6 (left side) and antiproliferative effects on HL60 cells (right side).

Compound	IC_50_ (µM) ± SE or Inhibition (%) @ Concentration (µM)		GI_50_ [µM] ± SE
	HDAC1	HDAC6	
**Bufexamac (8)**	31.66 ± 4.75	2.24 ± 0.05	31.78 ± 0.57
**9**	49 @ 300	13.72 ± 0.16	33.99 ± 0.06
